# Advancements in plant-derived exosome-like vesicles: Versatile bioactive carriers for targeted drug delivery systems

**DOI:** 10.1016/j.jpha.2025.101300

**Published:** 2025-04-14

**Authors:** Haixia Shen, Shuaiguang Li, Liyuan Lin, Qian Wu, Zhonghua Dong, Wei Xu

**Affiliations:** a School of Pharmacy, The Shandong Second Medical University, Weifang, Shandong, 261053, China; b Department of Clinical Pharmacy, The First Affiliated Hospital of Shandong First Medical University & Shandong Provincial Qianfoshan Hospital, Jinan, 250014, China; c Department of Oncology, The First Affiliated Hospital of Shandong First Medical University & Shandong Provincial Qianfoshan Hospital, Shandong Lung Cancer Institute, Jinan, 250014, China

**Keywords:** Plant-derived exosomes, Delivery of drugs, Extracellular vesicles, Nanocarriers, Nanoparticles

## Abstract

Exosomes, small vesicles secreted by a wide range of cells, are found extensively in animals, plants, and microorganisms. Their excellent biocompatibility, efficient delivery capacity, and ease of membrane crossing have drawn significant interest as promising drug delivery carriers. Compared with their animal-derived counterparts, plant-derived exosomes (PDEs), in particular, stand out for their lower toxicity to human tissues, diverse sources, and enhanced targeted delivery capabilities. Advances in both in-depth research and technological development have enabled scholars to isolate exosomes successfully from various plants, exploring their potential in clinical therapies. However, the precise identification of PDEs and their drug delivery mechanisms remains an area of ongoing investigation. This review synthesizes the latest developments in the biogenesis, extraction, separation, and identification of PDEs, along with their engineering modifications and drug-loading strategies. We also delve into the therapeutic applications of exosomes and their future potential in drug delivery, aiming to elucidate the targeted delivery mechanisms of PDEs and pave new paths for clinical drug treatment.

## Highlights

• Plant-derived exosomes are less likely to cause immune reactions and are biocompatible.
• Plant-derived exosomes are ideal carriers for drug delivery and gene therapy.
• Plant-derived exosomes can precisely regulate their loadings to meet different application requirements.

## Graphical abstract

Plant-derived exosomes, isolated through classical differential centrifugation, exhibit a broad spectrum of physiological activities. Utilizing these exosomes for drug delivery enables precise targeting of lesions, thereby enhancing therapeutic outcomes. By Figdraw.

**Figure:**
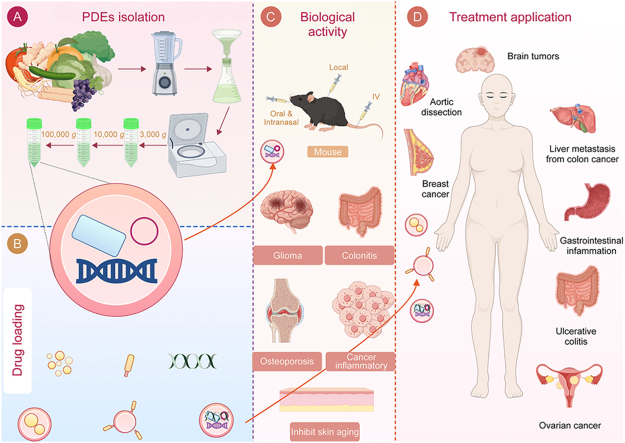

## 1 Introduction

Extracellular vesicles (EVs) are small, spherical, membrane-bound particles surrounded by a lipid bilayer [1]. Almost all life forms secrete EVs into their environment, where they serve as crucial mediators of intercellular communication and participate in diverse physiological processes. EVs are classified into three types based on their mechanism of biogenesis [2]. Exosomes, with diameters of approximately 30–150 nm, are released into the extracellular environment through the fusion of multivesicular bodies (MVBs) with the plasma membrane. Microvesicles, ranging from 100 to 1,000 nm in diameter, are directly released via exocytosis from the plasma membrane. Apoptotic vesicles, which are larger than 1,000 nm, are formed during apoptosis as a result of cell shrinkage and fragmentation. Exosomes and microvesicles carry RNA, DNA, proteins, and other biomolecules from their parent cells, with partially overlapping size ranges that complicate their differentiation. In contrast, apoptotic vesicles, which contain organelles and are larger in size, are more easily distinguishable [3]. Exosomes have distinct advantages over microvesicles and apoptotic vesicles in the fields of cellular communication and disease therapy because of their unique size, origin, function, biodistribution properties, and superior biosafety.

**Fig. 1:**
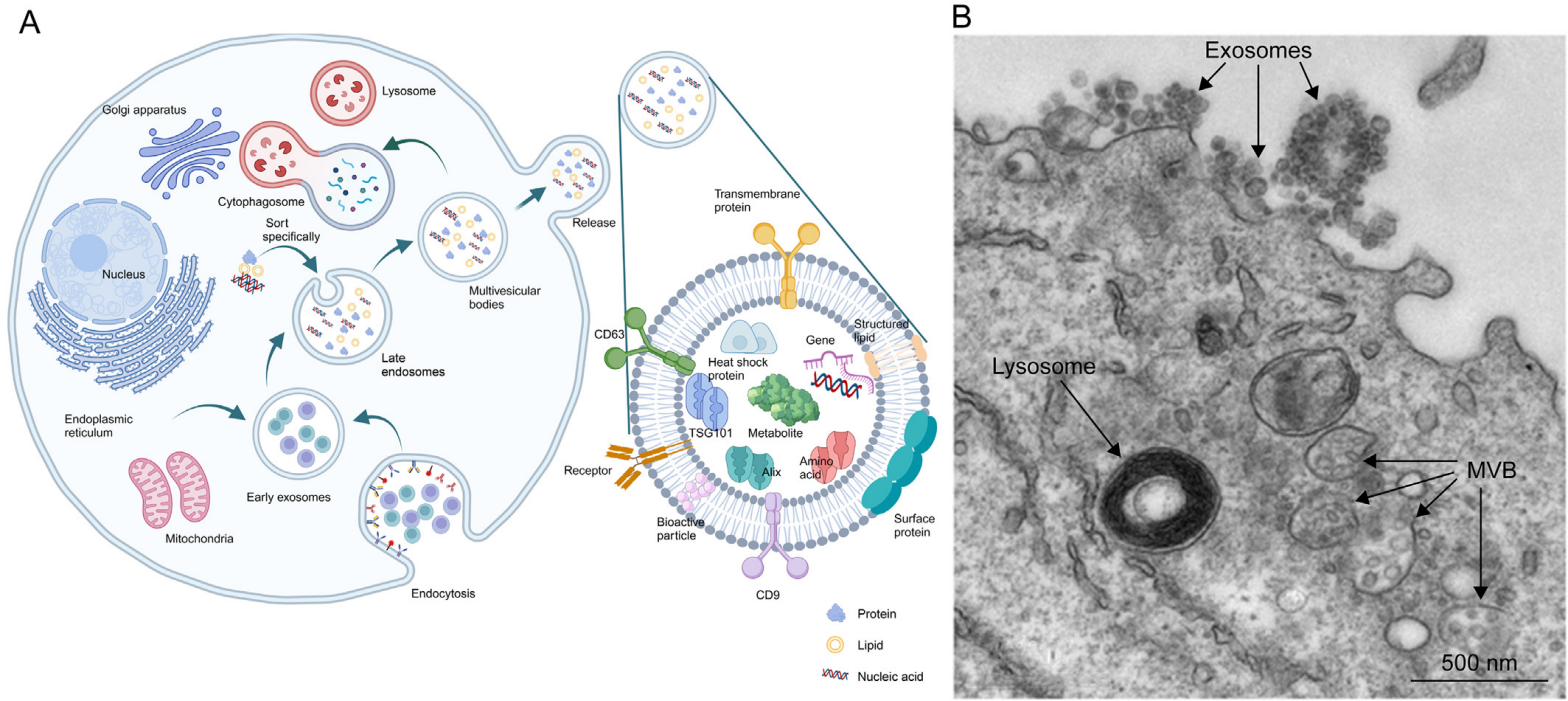
Exosome biogenesis and secretion pathway. (A) Biogenetic pathway and main composition of the exosomes. The formation of exosomes begins with endocytosis, which starts from invagination of the plasma membrane, leading to the generation of early endosomes. These endosomes progress to late endosomes, where the intraluminal vesicles mature into exosomes. The composition of exosomes is intricate, featuring surface markers such as CD63 and CD9, along with internal components, including nucleic acids, proteins, and lipids. Exosomes are subsequently released into the extracellular environment, where they are prepared for intercellular communication. Prepared using BioRender. (B) A transmission electron microscopy image of exosomes on the plasma membrane of a cell. An Epstein–Barr virus-transformed B-cell displaying newly expelled exosomes at the plasma membrane. Multivesicular bodies (MVBs) can be seen and can deliver their contents to the lysosomes for degradation or can fuse with the cell surface to release intraluminal vesicles as exosomes, as indicated by the *arrows* at the top of the picture. Reprinted from Ref. [10] with permission.

Exosomes are a type of EV characterized by a diameter of 30–150 nm. These vesicles facilitate intercellular communication and the transport of macromolecules. Exosomes are also involved in transporting proteins, lipids, mRNAs, miRNAs, and DNA, potentially contributing to the progression of various diseases [[4], [5], [6]]. Their composition of natural cell membranes, rather than synthetic polymers, ensures better biocompatibility with the host. Exosomes are secreted under both physiological and pathological conditions by nearly all cell types, positioning them as exceptional carriers for drug delivery [7,8]. Originating within cells, exosomes are released from the cell membrane and MVBs, encompassing multiple vesicles formed through extracellular fusion with the cell membrane [9] (as shown in Fig. 1A). First, it enters the cell through endocytosis, and the plasma membrane invaginates together with cell surface proteins and buds at the luminal side of the cell to form early sorting endosomes (ESEs), which in turn can further form late sorting endosomes (LSEs). The LSEs are filled with various inclusions (proteins, nucleic acids, lipids, etc.); therefore, when the LSEs membrane invaginates, the randomly wrapped inclusions are mixed to form a number of intraluminal vesicles (ILVs). The remaining membrane of the invaginated LSEs membrane serves as the outer membrane, which concentrates randomly wrapped inclusions in the LSEs lumen, further forming intracellular MVBs. Some MVBs can fuse with autophagosomes; eventually, their contents can be degraded in lysosomes, and the degradation products can be recycled by the cells. Other MVBs can also be transported to the plasma membrane through the cytoskeleton and microtubule network, fused with the plasma membrane with the help of MVB docking proteins, and secreted outside the cell into exosomes by cytosolic action [10] (as shown in Fig. 1B).

The excellent biocompatibility and low immunogenicity of exosomes suggest their potential use for drug delivery. Compared with traditional chemical drugs, which often suffer from poor biocompatibility, rapid clearance by the human body, and suboptimal solubility and permeability [11], exosomes offer multiple advantages: nanoscale size, low cytotoxicity, efficient membrane permeability, and biocompatibility. Furthermore, their lipid bilayer structure can protect the contents from degradation, preserving their biological activity [12]. As such, exosomes can serve as carriers to deliver drugs, facilitating their evasion of the immune system and crossing tissue barriers, thus increasing the efficiency of drug delivery and absorption. In particular, when exosomes are derived from a patient's own cells or certain types of cells, such as immature dendritic cells and mesenchymal stem cells, they exhibit almost no immunogenicity, which is beneficial for increasing the efficacy of the drug and reducing adverse reactions [13]; exosomes contain specific phospholipids and functional membrane proteins, enabling them to deliver their contents efficiently into the cytoplasm of target cells. Exosomes can bypass the endosomal–lysosomal pathway by mechanisms such as membrane fusion or gap junctions, which enhances delivery efficiency [14]; exosomes naturally possess targeting properties to some extent, reducing off-target effects and associated adverse reactions [15]. Currently, drug treatments for tumors focus primarily on eliminating tumor cells and inducing effective antitumor immune responses. Improving the stability, targeting, and delivery efficiency of drugs remains a significant challenge.

Exosomes are commonly classified based on their origin into animal-derived exosomes and plant-derived exosomes (PDEs). Animal-derived exosomes primarily originate from somatic cells, including tumor and immune cells, and play critical roles in tumor progression and immune regulation. For example, Hyung et al. [16] showed that patient-derived exosomes promote the targeting of oncogenic receptor tyrosine kinase (MET) in advanced gastric cancer and enhance antitumor effects. In addition, Wang et al. [17] found that M2 exosomes act as immunomodulators to promote diabetic fracture healing. Although the prospects for the application of animal-derived exosomes are broad, several major issues limit their clinical application, including low yields, laborious and time-consuming production processes, and difficulty in preparing high-quality exosomes with consistent uniformity [18,19]. PDEs, which are secreted by plant cells, have a composition similar to that of animal-derived exosomes but differ in specific components, conferring unique biological properties and potential therapeutic applications [20].

## 2 Composition and function of PDEs

Compared with animal-derived exosomes, PDEs have advantages, including a broader range of raw materials, ease of mass production, and lower extraction costs, indicating their vast potential applications in biomedicine [21,22]. Emerging evidence suggests that plant EVs play a crucial role in cross-kingdom molecular regulation with interacting organisms [23]. In addition to carrying signaling molecules (nucleic acids, proteins, metabolic wastes, etc.) for cell communication, plant EVs can also operate within the cell microenvironment across cellular boundaries [24].

While many similarities exist between PDEs and their animal counterparts, significant differences are also present between these two groups of vesicles. The lipid bilayer of animal-derived exosomes is primarily composed of cholesterol, glycosphingolipids, ceramides, and phosphatidylserines, which provide stability and unique resilience [[25], [26], [27]]. In contrast, the membranes of PDEs are rich in phosphatidic acid (PA), phosphatidylcholine (PC), digalactosyldiacylglycerol (DGDG), and monogalactosyldiacylglycerol (MGDG), and these distinct lipid traits confer inherent modulatory activities to plant cells [28]. PA, in particular, is prevalent in PDEs and has the ability to target and stimulate the rapamycin (mTOR) pathway, which is responsible for cell growth, proliferation, and recovery and is implicated in various human health and disease processes [29,30]. Teng et al. [31] isolated ginger exosome-like nanoparticles (GELNs) and analyzed their genetic, protein, and lipid profiles. GELNs, which are rich in phospholipid membranes, prioritize microbial uptake and can regulate the gut bacterial environment. Moreover, the presence of specific proteins and genes indicates that GELNs can modulate the gut microenvironment. In summary, the unique features of PDEs may facilitate interspecies communication.

## 3 Extraction and separation of PDEs

Compared with animal-derived exosomes, PDEs are simpler to produce. They can be obtained through various methods, including plant cell culture, extraction, and purification. Moreover, PDEs offer advantages such as abundant availability, scalability for mass production, and superior biosafety. Furthermore, plant cell culture conditions are relatively straightforward and easy to regulate, ensuring the stable and controlled production of PDEs.

**Fig. 2:**
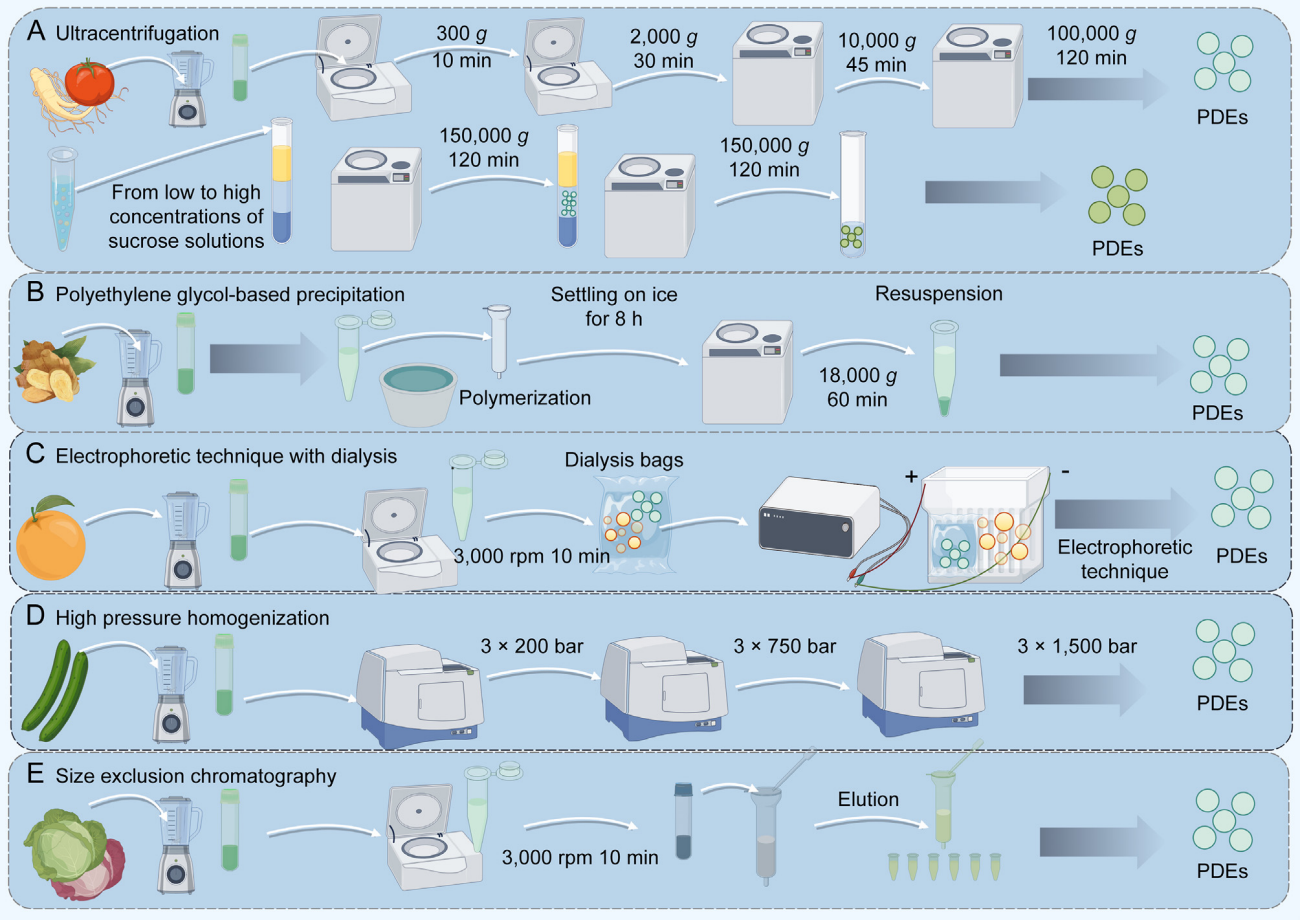
Processes for isolating plant-derived exosomes (PDEs). (A) Ginseng and tomato-derived exosomes were obtained by ultracentrifugation and purified by a sucrose gradient [39,40]. (B) Ginger-derived exosomes were obtained by polyethylene glycol precipitation [41]. (C) Grapefruit-derived exosomes were obtained by electrophoretic dialysis [42]. (D) Cucumber-derived exosomes were obtained by high-pressure homogenization [43]. (E) Cabbage-derived exosomes were obtained by size exclusion chromatography [44]. Prepared using Figdraw.

PDEs are receiving increasing attention for their potential roles in human health and drug delivery. The isolation and purification of these exosomes are critical first steps in investigating their functions and mechanisms [32]. The development of rapid and efficient methods that maintain the stability, integrity, and biological activity of exosomes remains a significant barrier to their clinical translation [33]. Ultracentrifugation is the most widely used method for exosome extraction, exploiting the differences in volume and density between exosomes and other components to sediment them at different centrifugation speeds. This process encompasses three steps: low-speed centrifugation (300 *g*) to clear dead cells, medium-speed centrifugation (2,000 *g*) and high-speed centrifugation (10,000 *g*) to remove cell debris, and ultrahigh-speed centrifugation (100,000 *g*) to precipitate and concentrate the exosomes [34]. Sucrose gradient centrifugation, a more refined form of ultracentrifugation, further separates vesicles of different densities, particularly exosomes [35]. In addition to sucrose gradients, newer isopycnic centrifugation methods, such as iodixanol gradients, better preserve the biophysical properties of vesicles. While ultracentrifugation-based methods are straightforward and cost-effective, they are time-consuming, require expensive equipment, and often result in poor purification specificity and reduced biological activity. Additionally, high centrifugal forces can lead to the aggregation of exosomes, making their large-scale application challenging [36]. Additionally, density gradient centrifugation enables the collection of EVs based on their buoyant density and is commonly employed to isolate specific fractions of EVs [37]. This method creates a continuous or stepwise gradient on a sucrose matrix [38]. For example, ginseng nanovesicles and tomato-derived nanovesicles have been isolated from ginseng root homogenate using differential centrifugation and sucrose density gradient techniques (Fig. 2A) [[39], [40]].

**Fig. 3:**
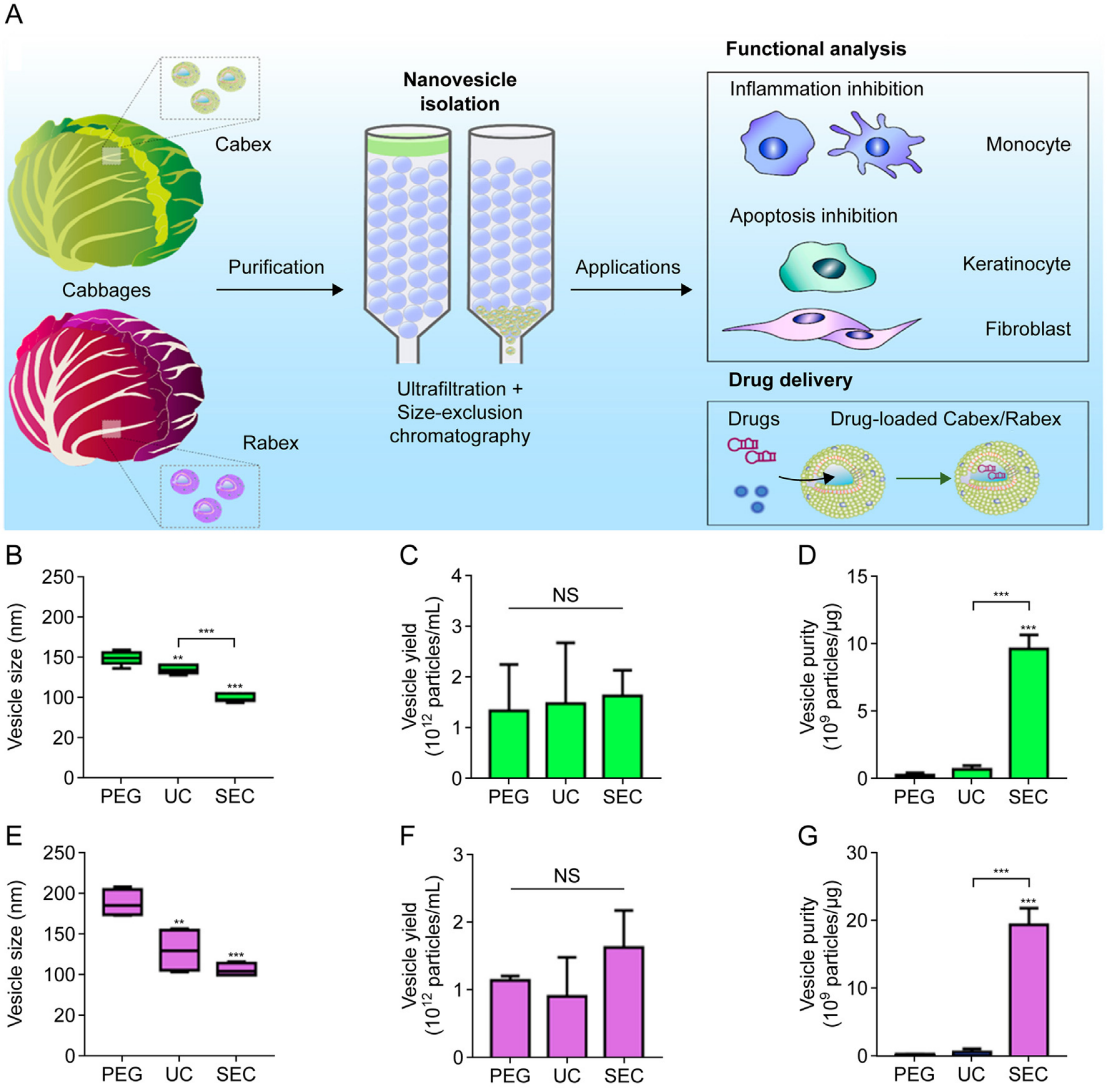
Comparison of nanovesicles isolated from Chinese cabbage and red cabbage by different methods. (A) Schematic illustration of exosome-like nanovesicle isolation from cabbage and the investigation of molecular functions (inflammation and apoptosis inhibition) and applications (drug delivery) of Cabex and Rabex. (B–D) Comparison of average size (B), EV yield (C), and nanovesicle purity (D) of Cabex between isolation methods. (E–G) Comparison of average size (E), nanovesicle yield (F), and nanovesicle purity (G) of Rabex between isolation methods (Green represents cabbage, and pink represents red cabbage) All values are expressed as mean ± standard deviation (SD) (^∗∗^*P* < 0.01; ^∗∗∗^*P* < 0.001; NS: not significant; *n* = 3). PEG: polyethylene glycol precipitation; UC: ultra-centrifugation; SEC: size exclusion chromatography. Reprinted from Ref. [44] with permission.

Polyethylene glycol precipitation has also been used to extract and isolate ginger-derived exosomes (Fig. 2B) [41]. This method leverages the principle that particles of varying sizes traverse a gel column at different rates, enabling the separation of exosomes from diverse samples. Currently, single extraction methods often yield PDEs with limited reproducibility, poor homogeneity, and reduced stability. Consequently, researchers have adopted combinatorial approaches involving two or more extraction and isolation methods to achieve highly pure and homogeneous PDEs. Additionally, electrophoretic dialysis [42] and high-pressure homogenization [43] have also been employed for the extraction of PDEs (as shown in Figs. 2C and D). Another efficient method for the extraction and purification of PDEs is size exclusion chromatography (SEC) (Fig. 2E) [44]. You et al. [44] isolated high-purity vesicles from cabbage and red cabbage using a combination of these two methods and compared their properties with those of vesicles obtained via ultracentrifugation, polyethylene glycol-based precipitation, and size-exclusion chromatography. As illustrated in Fig. 3, nanovesicles isolated via ultracentrifugation and polyethylene glycol-based precipitation presented multiple peaks with average sizes of 134.2 and 148.2 nm, respectively, suggesting the presence of heterogeneous nanoparticles. In contrast, nanovesicles obtained using differential centrifugation combined with size-exclusion chromatography presented a relatively homogeneous peak. Notably, the yield remained comparable across all three methods.

The emergence of novel means of extraction and isolation has also been driven by the limitations of traditional method. Methods such as ultrafiltration (UF), immunoisolation, microfluidic technology, ion exchange chromatography (IEC), and membrane separation techniques have been developed to isolate EVs based on size, sedimentation, and immunological affinity [45]. Nevertheless, these methods are rarely applied to the extraction of PDEs. Therefore, advancing the development and application of PDEs requires the exploration of novel isolation techniques, leveraging emerging strategies from animal-derived exosome extraction while accounting for the unique vesicular properties of PDEs. Despite ongoing improvements in techniques for isolating plant EVs, their rapid, efficient, reproducible, and large-scale separation remains a challenge. Table 1 summarizes the novel technologies used for the extraction and separation of exosomes [[46], [47], [48], [49], [50], [51], [52], [53]].

**Table 1:** New techniques for the extraction and isolation of exosomes.

Method	Principle	Advantages	Disadvantages	Refs.
Ultrafiltration (UF)	Utilizes membrane filters and pressure to remove contaminants of larger molecular size.	Rapid, no need for expensive equipment, scalable for large-scale use.	Inability to separate exosomes from associated proteins.	[46,47]
Immunoisolation (Immunoaffinity)	Based on surface biomarkers, separation is achieved by coating antibodies on the surface of magnetic beads.	High purity of separation.	Requires additional steps for separation and purification, not suitable for large-scale applications.	[48]
Microfluidic technology	Separates exosomes using their physical and biochemical characteristics at a microscale.	High purity, high efficiency.	Not suitable for large-scale applications, primarily used for diagnostic purposes.	[[49], [50], [51]]
Ion exchange chromatography (IEC)	Negatively charged EVs bind to positively charged chromatographic columns and are separated by increasing the ionic strength of the mobile phase.	Easy to apply.	Non-specific separation.	[52]
Membrane separation techniques	Specific binding of hydrophilic phosphate groups on the surface to certain metal oxides (TiO_2_, ZrO_2,_ etc.) or separation based on surface charge differences.	Effective for separating larger volume exosomes.	Susceptible to contamination by other molecules with similar membrane properties.	[53]

EVs: extracellular vesicles; TiO_2_: titanium dioxide; ZrO_2_: zirconium (IV) oxide.

Typically, vesicle isolation methods should maintain the vesicle integrity while possessing sensitivity and specificity. Cost-effective, rapid, and simple methods that preserve the vesicle integrity should be capable of producing high quantities of highly pure vesicles. However, current exosome isolation methods are costly and time-consuming. Hence, the development of rapid and low-cost technologies is needed to meet the demand for large-scale separation of high-purity PDEs [54]. Moreover, many common exosome purification methods fail to remove degraded particles or soluble substances associated with exosomes, potentially leading to biological side effects. *In vitro* and *in vivo* studies must adhere to reliable purification schemes and safety protocols to minimize these contaminants. Therefore, these issues should be addressed by optimizing existing exosome isolation techniques or employing multiple methods concurrently [55,56].

## 4 Characterization and identification of PDEs

The size, morphology, concentration, surface charge, and structure of PDEs are characterized using various analytical techniques. Nanoparticle tracking analysis (NTA) is commonly employed to measure the size and concentration of particles between 10 and 2,000 nm, whereas dynamic light scattering (DLS) provides size information but is more suitable for high-concentration samples, detecting PDE diameters typically between 50 and 500 nm with zeta potentials ranging from −25 to −15 mV [57]. Flow cytometry can detect larger particles above 500 nm or smaller particles when fluorescent markers are used [58]. Transmission electron microscopy (TEM) and scanning electron microscopy (SEM) provide detailed insights into PDE morphology, which often results in uniform spherical structures, although sample preparation processes can sometimes create a cup-shaped appearance. Specific examples of PDE sizes include ginseng-derived exosomes at an average of 344.8 nm [58], ginger-derived exosomes at 188.5 nm [59,60], and yam-derived exosomes with two populations averaging 168 and 328 nm [61]. Similar to plant MVBs, PDEs from a single plant tissue or organ may contain multiple particle subtypes. Grape-derived exosomes have two distinct peaks at 31.64 ± 5.00 nm and 139.27 ± 14.11 nm, accounting for 47% and 35% of the mass, respectively [62].

Differences in extraction and isolation methods may lead to changes in some vesicle characteristics of PDEs, such as the particle size, size uniformity, or changes in the zeta potential, but do not have much effect on their morphology, and thus the morphology of PDEs remains the gold standard for identification and characterization. Although electron microscopy (EM) allows the direct visualization of particle morphology and size, sample preparation involving fixation and dehydration often results in the cup-shaped morphologies observed via SEM and TEM [63]. Currently, most PDEs are identified by TEM, nanoparticle tracking analysis, and protein markers; however, no accurate standard protein is available for identifying plant-derived exosome surface markers. This lack of standardization poses challenges for future research on PDEs. Therefore, establishing a clear identification standard for PDEs is crucial for their further application in the medical field.

## 5 Behavioral mechanisms and biological functions of PDEs

**Fig. 4:**
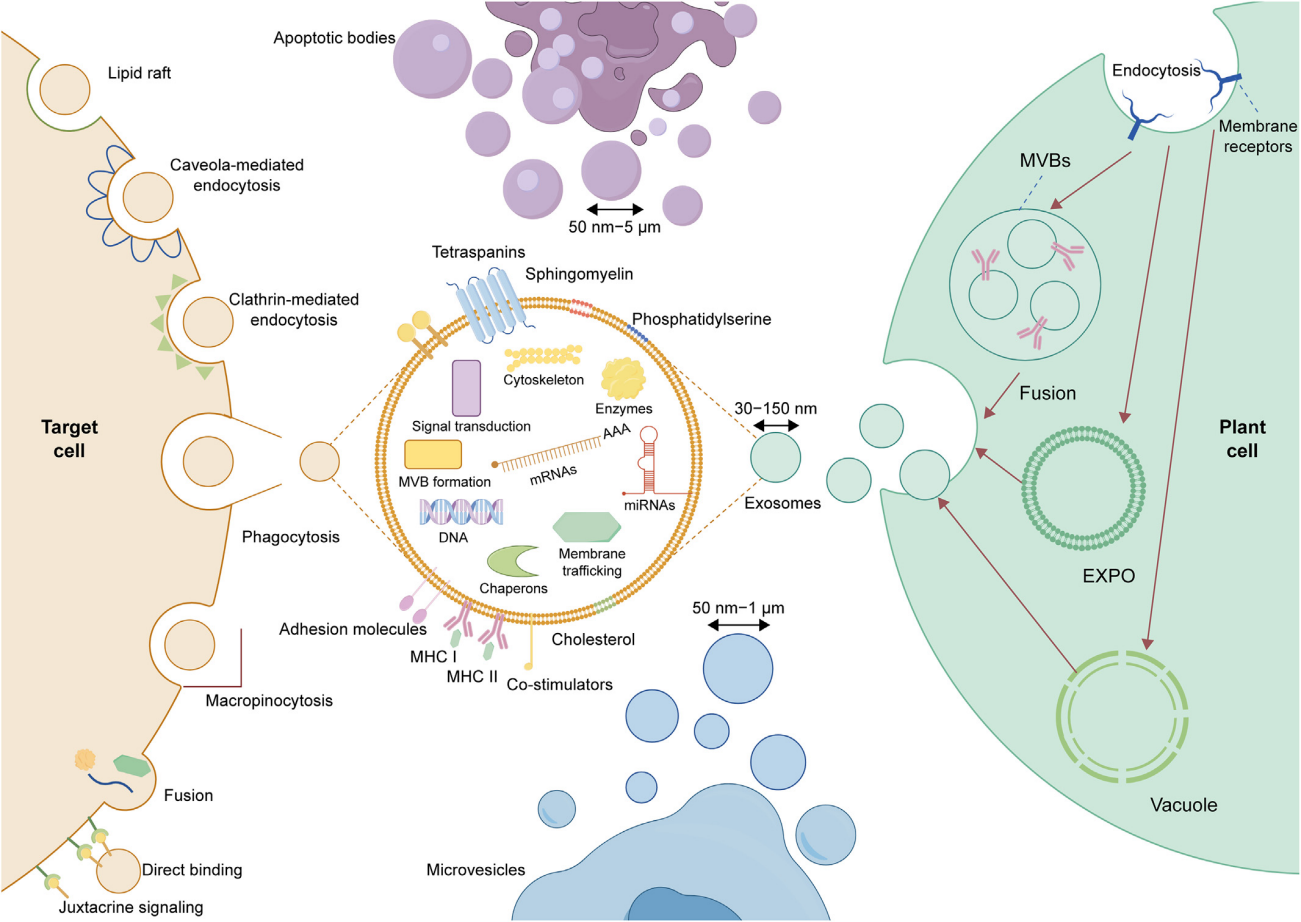
Biogenesis pathways of plant-derived exosomes (PDEs) and targeting modes. PDEs are secreted by plant cells through three distinct pathways, feature a natural lipid bilayer structure on their surface, and contain various proteins and nucleic acids inside. These exosomes enter target cells to exert physiological effects through direct binding to receptors, membrane fusion, or endocytosis. MVBs: multivesicular bodies; EXPO: exocyst positive organelles; MHC: major histocompatibility complex. Prepared using Figdraw.

The biogenesis of PDEs is characterized by a main pathway identical to that of animal exosomes and two additional pathways (as shown in Fig. 4) [64,65]. In the exocyst positive organelles (Expo) pathway, the double-membrane structure of Expo is formed inside the cell and then fuses with the plasma membrane, releasing single-membrane vesicles into the extracellular environment in the form of EVs [66,67]. In the vesicular pathway, MVBs first release their contents into vesicles and then further release the vesicles into the extracellular environment by fusing the vesicles with the plasma membrane [68,69].

PDEs are widely recognized to enter target cells through three primary mechanisms (as shown in Fig. 4) [70,71]: direct fusion with the target cell membrane, enabling the release of cargo into the cytoplasm [72]; endocytosis, where PDEs enter the target cell and subsequently release their cargo into the cytoplasm [73], which involves common endocytic pathways, such as clathrin-mediated endocytosis [74], and lipid raft-associated membrane invagination [75], and mediates exocytic internalization [76]; and receptor binding, where PDEs interact with specific receptors on the target cell membrane, initiating receptor‒ligand interactions and downstream signaling cascades to activate the target cell [77]. An in-depth understanding of the biosynthetic pathways of PDEs and the mechanisms underlying their interactions with receptor cells lays the foundation for optimizing their therapeutic applications. Precise regulation of the generation process and endocytosis pathways, along with enhancing their selective affinity for specific target cells, can significantly improve the efficiency and accuracy of drug delivery. In conclusion, refining the engineered design of PDEs will pave the way for the development of more effective and safer drug delivery systems, thereby advancing the field of precision medicine.

**Fig. 5:**
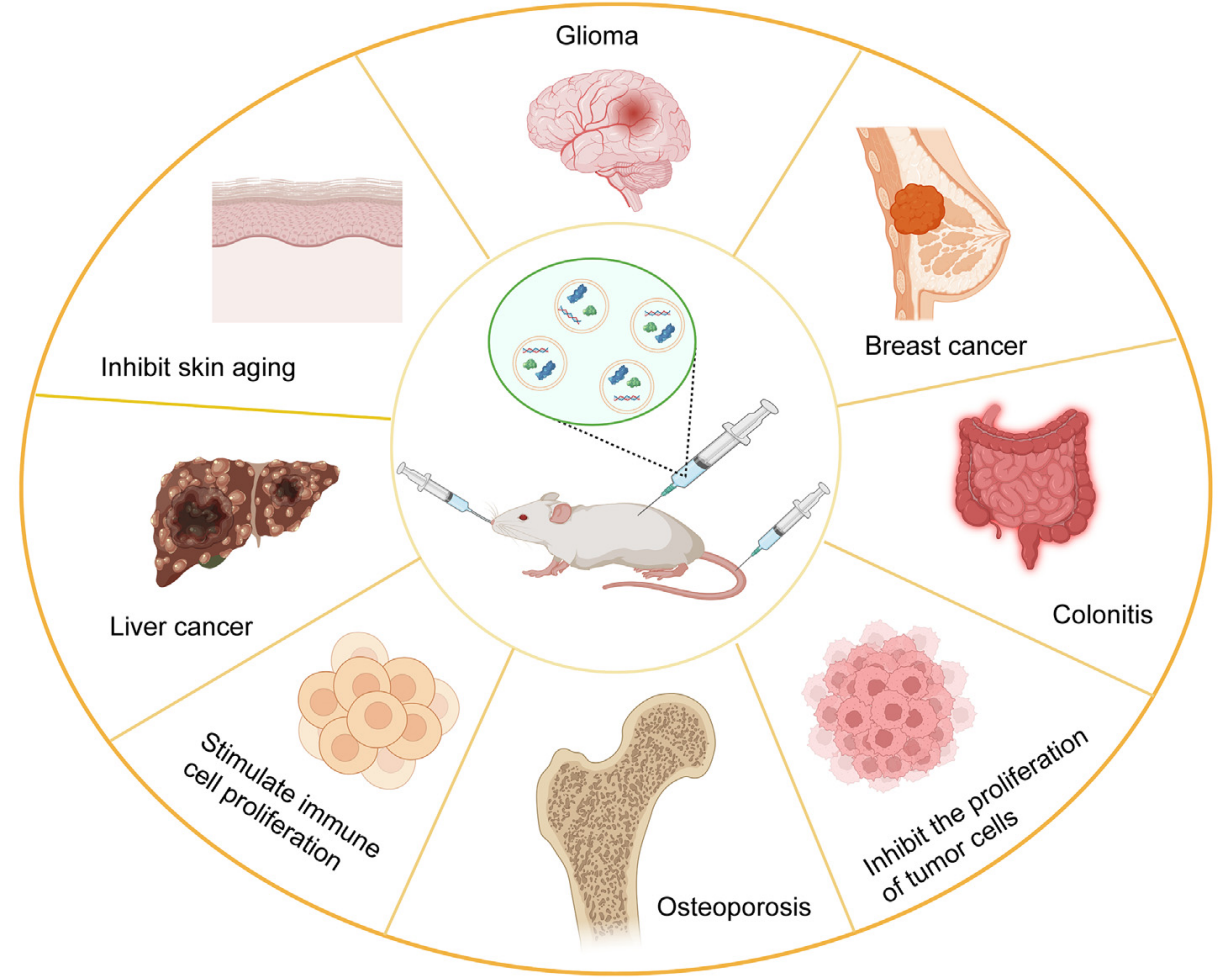
Therapeutic applications of plant-derived exosomes (PDEs). PDEs can exert pharmacological effects on various diseases when injected into mice. For example, they have been used to treat glioma, liver cancer, and breast cancer, inhibit skin aging, treat osteoporosis, stimulate immune cell proliferation, and ameliorate colitis.

Fresh plants, including herbs, seeds, vegetables, fruits, and their extracts, are rich sources of diverse nutrients, such as vitamins, minerals, fibers, proteins, and other health-beneficial components. Moreover, they have been associated with reduced risks of cancer, chronic diseases, and inflammation [78]. Consequently, plant biomolecules have garnered significant interest for their potential to improve health and prevent a variety of diseases. Some natural bioactive compounds extracted from medicinal plants play pivotal roles in the clinical treatment of various diseases. Due to the cellular origin of PDEs, each PDE has distinct characteristics and compositions, leading to diverse functions. PDEs and their inherent molecules demonstrate a range of functionalities through various mechanisms and cellular uptake and are increasingly applied in the development of new drugs targeting specific diseases or maintaining healthy bodily functions [79]. A variety of edible plants have been utilized to isolate therapeutic exosomes with multifunctional properties (as shown in Fig. 5). Table 2 lists several important plants used to extract PDEs and their therapeutic applications [[80], [81], [82], [83], [84], [85], [86], [87], [88], [89], [90], [91], [92]].

**Table 2:** Important sources of plant-derived exosomes (PDEs) and their therapeutic applications.

Plant	Average diameter (nm)	Site of action	Biological activity	Refs.
Ginseng	151.6	Glioma	Regulates cell proliferation and modulates the tumor microenvironment (TME) with significant tumor growth inhibition	[80]
Ginger	132	Various human sites	Anti-inflammatory, shapes gut microbiota, antiviral	[81,82]
Sanghuang	100–260	Skin	Inhibits mical 2 expression in HaCaT cells, promotes COL1A2 expression, suppresses MMP1 in skin cells, reduces ROS, MDA, and SA-β Gal levels, and enhances UV-induced SOD activity, inhibiting skin aging.	[83]
Purslane	160	Colon	Promotes the proliferation of double-positive CD4^+^CD8^+^ T cells to alleviate DSS-induced colitis.	[84]
Tea	166.9	Breast tumor	Directly induces apoptosis and modulates microbiota for effective treatment against breast tumors.	[85]
Asparagus	92; 119; 179	Liver cancer cells	Inhibits proliferation of liver cancer cells, induces apoptosis, and upregulates apoptotic factors.	[86]
Chives	145 ± 2	Microglia	Affects LPS-stimulated BV-2 and MG-6 cells and inhibits the expression of inflammatory mediators.	[87]
Garlic	144.95; 133.58	Type 2 diabetes	Trained human gut Akkermansia muciniphila can reverse high-fat diet-induced type 2 diabetes (T2DM) in mice.	[88]
Momordica charantia L.	155	Doxorubicin cardiotoxicity	Stabilizes p62 expression to ameliorate doxorubicin cardiotoxicity.	[89]
Lepidium meyenii Walp (Maca)	134	Depression	Ameliorates depression by promoting 5-HT synthesis via the modulation of gut–brain axis.	[90]
*Brassica oleracea*	115.2	Colitis-associated inflammation	The suppression of inflammation in macrophages and promotion of colon epithelial cell regeneration.	[91]
Gouqi	127.8 ± 1.3	Dexamethasone-induced muscle	Improves the quality and function of skeletal muscle accompanying the activated AMPK/SIRT1/PGC1α signaling pathway.	[92]

COL1A2: collagen type I alpha 2 chain; MMP1: matrix metallopeptidase 1; ROS: reactive oxygen species; MDA: malondialdehyde; SA-β Gal: senescence-associated β-galactosidase. SOD: superoxide dismutase; DSS: dextran sulfate; p62: sequestosome 1; AMPK: adenosine 5′-monophosphate (AMP)-activated protein kinase; SIRT1: silent mating type information regulation 2 homolog- 1; PGC1α: peroxisome proliferators-activated receptor γ coactivator 1-alpha.

## 6 Surface modification strategies for PDEs

PDEs, also known as exosome-like nanoparticles, selectively target human cells through a distinct endocytosis pathway to exert therapeutic effects [93]. Moreover, when engineered with specialized modifications, these PDEs can achieve extended circulation half-lives and enhanced stability, acquiring organ-targeting capabilities, although the targeting mechanisms remain to be elucidated. Researchers, both domestically and internationally, have employed bioengineering techniques to modify PDEs, increasing their targeted drug delivery functions [94]. Three common modification strategies exist: first, genetic engineering, which involves fusing the gene sequences of guiding proteins or peptides with those of selected exosomal membrane proteins; second, chemical modifications, which, though less explored, allow the exosome surface to present a wide range of natural and synthetic ligands via chemical methods; and third, membrane fusion techniques, where exosomes encapsulating specific membrane proteins isolated from genetically modified cells are fused with various liposomes. This method represents a novel strategy for rationally designed exosomes to serve as hybrid nanocarriers in advanced drug delivery systems [95].

### 6.1 Genetic engineering modifications

The genetic engineering of exosomes represents a convenient method to confer new properties upon them. Initially, ligands or homing peptides are fused with transmembrane proteins expressed on the surface of exosomes. Subsequently, engineered exosomes displaying targeting ligands are secreted by donor cells that have been transfected with plasmids encoding fusion proteins [96,97]. Currently, this approach is predominantly applied to animal-derived EVs, and surface modifications of PDEs are relatively rare. The use of genetic engineering to modify PDEs could become an innovative direction for future research, particularly for drug delivery systems.

### 6.2 Chemical modification

Chemical modification of plant-derived exosome surfaces is often employed for targeted drug delivery. For example, researchers have engineered exosome-like nanoparticles from ginger, which present folic acid as a ligand for arrow-tail RNA nanoparticles. This ligand facilitates the intravenous delivery of siRNA for tumor suppression, ultimately inhibiting the growth of human epithelial cancer cells [98]. Furthermore, researchers have developed folic acid (FA)-modified grapefruit-derived nanocarriers for the delivery of paclitaxel in cancer treatment. These nanocarriers exhibited a significantly improved targeting efficiency and effectively reduced the tumor volume in mice transplanted with SW620 or CT26 cells [99,100].

### 6.3 Membrane fusion

Membrane fusion is a technique employed to load therapeutic agents into exosomes. This membrane engineering approach represents an innovative strategy for generating hybrid exosomes as novel bio-nano transporters (BNTs) [101]. These engineered hybrid exosomes can transport exogenously incorporated hydrophobic lipids to recipient cells and hydrophilic cargo within exosomes as part of an advanced drug delivery system. In one study, fusion exosomes carrying the viral fusion protein vesicular stomatitis virus (VSV)-G directly delivered membrane proteins to target cells, providing new tools for membrane protein therapy. The fusion of exosomes with virus-like fusogenic vesicles (Vir-FVs) results in hybrid exosome formation [102]. Hybrid exosomes fused with liposomes have been used to deliver the CRISPR‒Cas9 system for targeted gene editing [103] and to increase the antitumor activity of cancer drugs [104]. In addition, studies have also been conducted to enhance the targeting of PDEs using membrane fusion techniques, for example, grapefruit-derived nanovectors (GNV) coated with inflammatoryrelated receptor enriched membranes of activated leukocytes (IGNVs), which significantly enhanced their aggregation at the site of inflammation and augmented their accumulation in target tissues [105].

## 7 Drug loading methods for PDEs

**Fig. 6:**
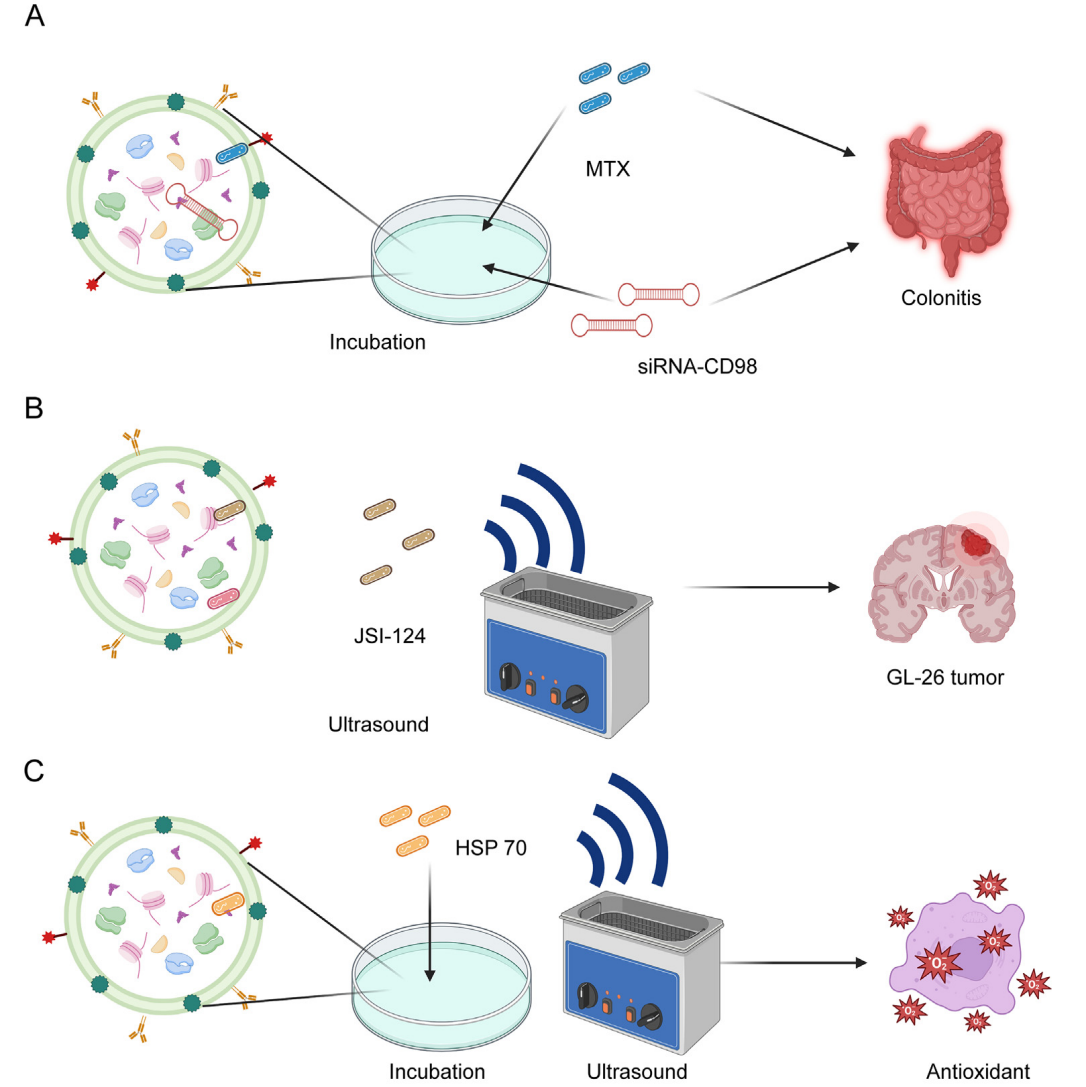
The main methods of drug encapsulation and applications of plant-derived exosomes (PDEs). (A) Passive loading through an incubation has been used to prepare ginger-derived extracellular vesicles (EVs) encapsulating siRNA-CD98 [111] and grapefruit-derived EVs encapsulating the anti-inflammatory drug methotrexate (MTX) for treating colitis [112]. (B) Active loading through ultrasonication has been used to prepare grapefruit-derived EVs carrying cucurbitacin I (JSI-124) to inhibit GL-26 tumor growth [113]. (C) The combination of passive and active loading entails an incubation followed by sonication, where EVs are used to load heat shock protein 70 (HSP 70) for its antioxidant properties [114]. The choice and innovation of loading methods are crucial for improving the encapsulation efficiency, loading rate, and stability of PDEs. Prepared using BioRender.

Compared with traditional nanocarrier systems, exosomes offer superior biocompatibility, greater utility, and nontoxicity and successfully carry model drugs and target genes for application in drug delivery systems for various diseases [106]. One advantage of PDEs is that they can be extracted from numerous edible plants, allowing for their efficient and abundant production. Due to these benefits, PDEs also hold promise as endogenous carriers for drug delivery [107]. Reports of animal exosomes loaded with chemical drugs, nucleic acid drugs, and plant extracts have been published [108,109]. Studies on the loading of cargoes by PDEs are relatively rare (as shown in Fig. 6) [[111], [112], [113], [114]]. However, due to their similarity to animal-derived exosomes, the drug loading methods used for animal exosomes may also be applicable to plant-derived exosomes (PDEs) [110]. Therefore, the development of efficient and convenient drug loading methods for PDEs is a key research area for advancing them as effective drug delivery systems.

Animal exosome drug loading can be categorized into two main pathways: the exogenous pathway, which involves extracting and purifying exosomes before loading them with therapeutic drugs, and the endogenous pathway, which involves drugs entering donor cells and being incorporated into exosomes that are then secreted and isolated as drug-laden exosomes. Two primary approaches are used for loading cargoes into PDEs: passive loading, such as sonication, and active loading, such as coincubation. Additionally, studies combining passive and active loading techniques have documented improved encapsulation rates. Currently, research methods for loading drugs into PDEs are limited. However, due to their similarity to animal-derived exosomes, the methods developed for animal exosomes may also be applicable to their plant-derived counterparts [115]. Therefore, developing efficient and convenient methods for loading drugs into PDEs is crucial for enhancing their potential as effective drug delivery systems [116]. Currently, techniques such as ultrasonic treatment, electroporation, transfection, incubation, extrusion, saponin-assisted loading, genetic modification, freeze-thaw cycles, heat shock, pH gradient, and hypotonic dialysis have been employed to load these drugs into exosomes. Table 3 summarizes the advantages and disadvantages of various exosome drug loading techniques [[117], [118], [119], [120], [121], [122], [123], [124], [125], [126], [127], [128], [129]].

**Table 3:** Drug loading methods of plant-derived exosomes (PDEs).

Method	Advantages	Disadvantages	Refs.
Extrusion	High drug loading efficiency	Alters the properties of the secreted exosomal membrane (e.g., protein structure, zeta potential).	[117]
Saponin-assisted loading	High drug loading efficiency	Saponins are difficult to remove completely, increasing exosome permeability and cytotoxicity.	[118]
Ultrasonic treatment	High efficiency in drug loading and sustained release	Can cause exosome aggregation and affect the structure of surface proteins.	[119]
Electroporation	Simple operation, widely applied	May cause RNA precipitation, affecting drug loading efficiency.	[[120], [121], [122]]
Transfection	High efficiency, good molecular stability	Potential toxicity and safety issues; may affect the nucleic acid drugs and biological activity.	[123]
Incubation	Simple operation, no need for other active substances	Lower encapsulation rate	[124,125]
Freeze-thaw cycles	Simple operation, mild conditions, rarely damages biological activity	Low drug loading rate	[126,127]
Heat shock	Does not affect exosome morphology and increases immunogenicity.	Affects membrane fluidity and drug stability.	[128]
pH gradient	High drug loading efficiency; does not affect the stability of nucleic acid drugs.	Reduces the total protein content of extracellular vesicles.	[129]

## 8 Applications of plant-derived exosome-like nanoparticles as drug delivery carriers

**Fig. 7:**
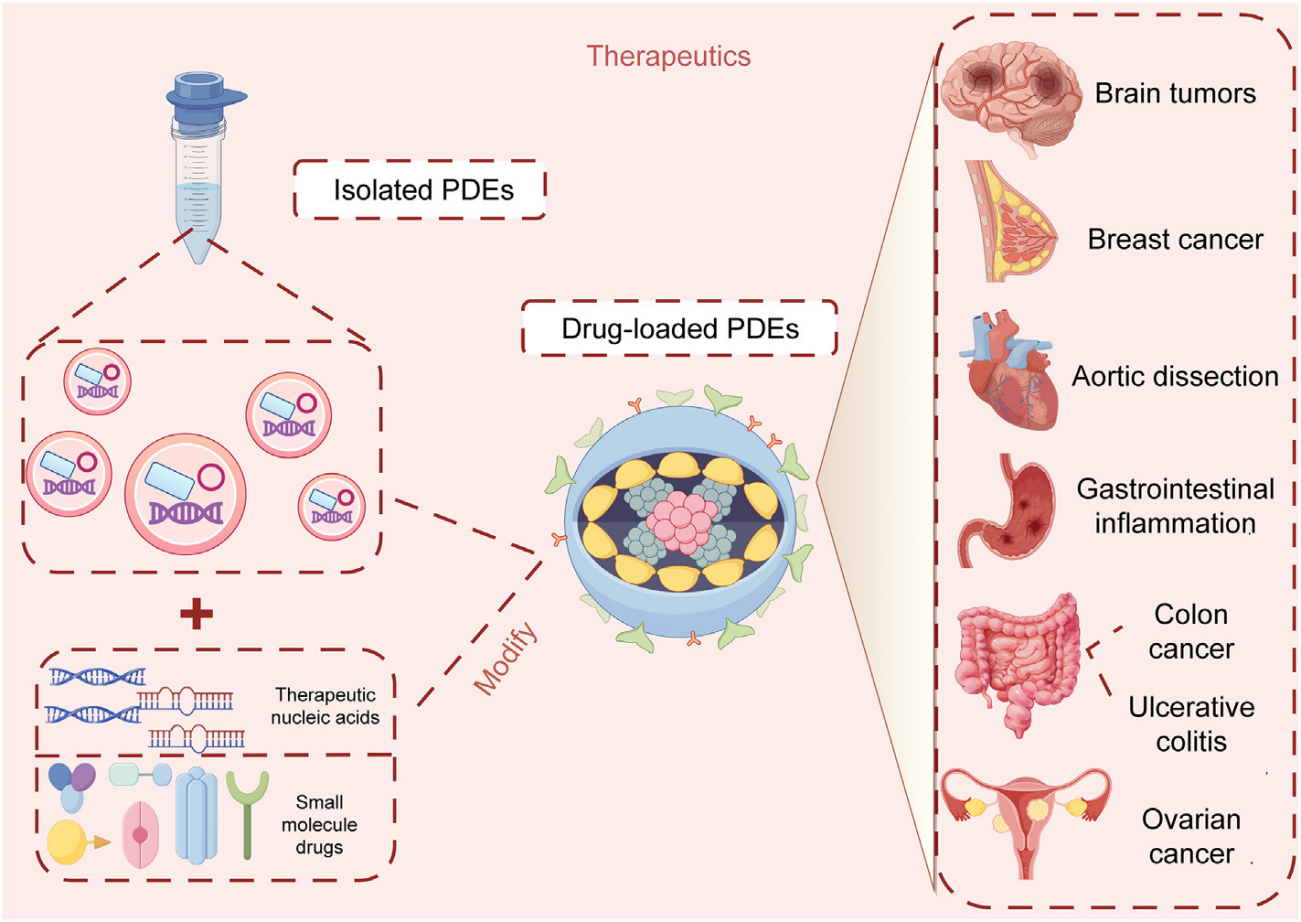
Mechanism of plant-derived exosome-mediated drug delivery. Plant-derived exosomes (PDEs) can be engineered to target specific disease sites by modifying the encapsulated drug. Upon reaching the body, they exert therapeutic effects on a range of conditions, including brain tumors, aortic coarctation, gastrointestinal inflammation, colon cancer, breast cancer, ulcerative colitis, ovarian cancer, and other metastatic liver diseases. Prepared using Figdraw.

The solubility of drug molecules directly affects the oral bioavailability and efficacy of medications. The use of plant-derived or animal exosomes as delivery systems allows direct oral intake with excellent biocompatibility, facilitating increased drug intake and accumulation within the body, thereby improving therapeutic outcomes. PDEs can also transport anticancer drugs, overcoming the limitations of bioavailability, stability, and safety posed by nonfood-derived exosomes and synthetic lipids [130]. Similar to artificial liposomes, lipid nanoparticles, and natural EVs, PDEs act as lipid carriers capable of loading and delivering drugs to target cells, thereby exerting therapeutic effects on the human body (as shown in Fig. 7).

According to current reports, two main types of drug delivery using PDEs have been identified, with direct drug loading and delivery being more frequently reported. For example, microRNAs (miRNAs), due to their biological activity, have a wide range of therapeutic applications. However, miRNAs are prone to rapid degradation when applied directly, necessitating the development of appropriate strategies to increase their therapeutic potential. Del Pozo-Acebo et al. [131] combined ultracentrifugation with size-exclusion chromatography to extract EVs from broccoli and then loaded miRNAs into these vesicles using incubation methods. The uptake of miRNAs by intestinal cell lines was detected, indicating that broccoli-derived EVs could increase the biological stability of the therapeutic RNA against ribonuclease degradation and gastrointestinal digestion. This property enhances their potential as drug delivery carriers. Furthermore, Wang et al. [112] incorporated the anti-inflammatory drug methotrexate (MTX) into grapefruit-derived EVs (GDNs) and delivered MTX-GDNs to mice. Compared with free MTX, this treatment significantly reduced MTX toxicity and increased its therapeutic effect on dextran sulfate (DSS)-induced colitis in mice. These findings suggest that GDNs can act as immune modulators in the gut, maintaining macrophage homeostasis, and could be developed for the oral delivery of small-molecule drugs to mitigate inflammatory responses in human diseases.

**Fig. 8:**
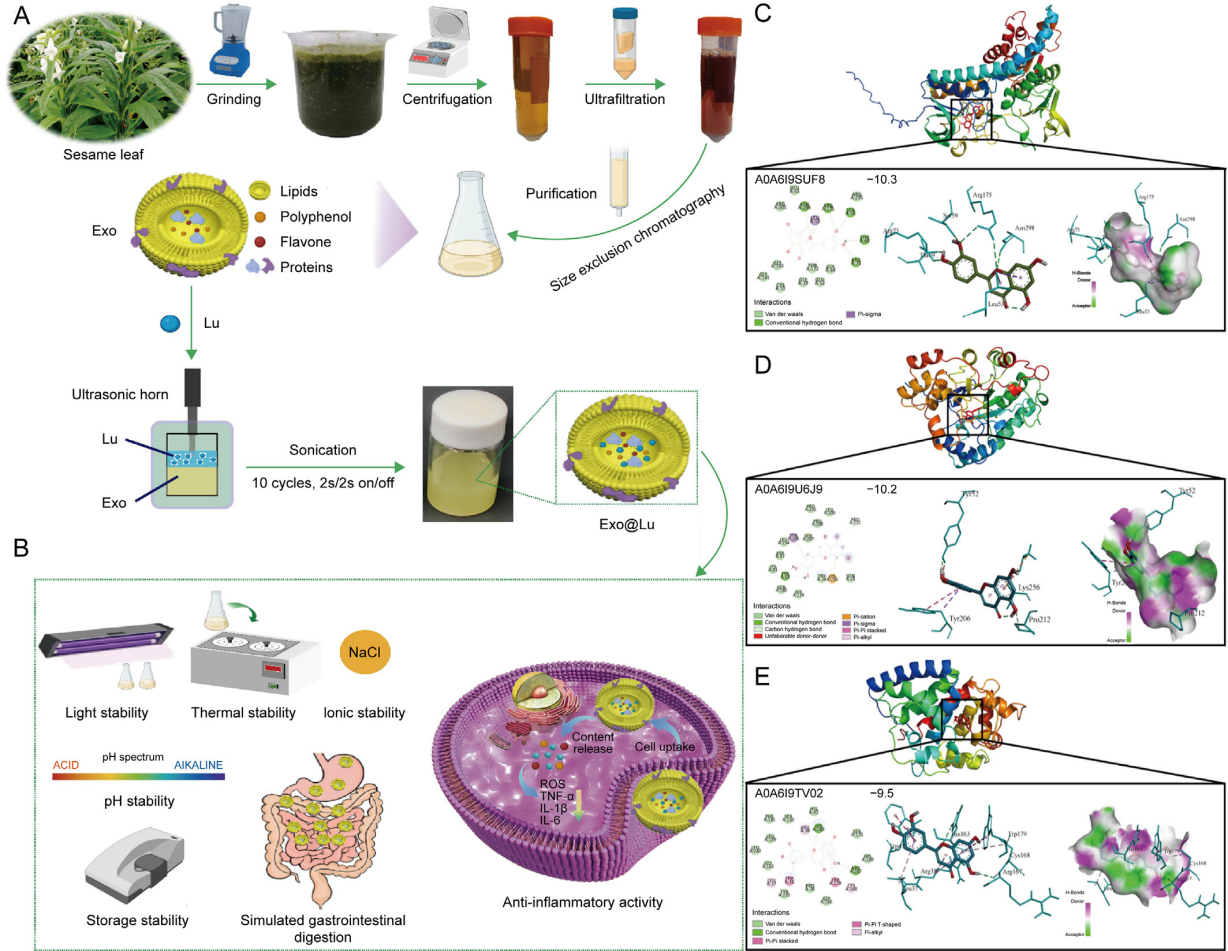
Schematic diagram of the process for preparing exosomes (Exo) nanoparticles and their application in Lu encapsulation. (A) The extraction and purification of Exos to encapsulate Lu. (B) Evaluation of the stability and anti-inflammatory activity of Exos@Lu. Molecular docking of Lu with Exos. (C) The 3D binding graph shows the optimal docking posture of the protein–Lu complex by visualization using PyMOL. Red: stick mode. (D, E) From left to right: 2D schematic diagram presenting the binding site and details of the amino acid residues of the protein interacting with Lu; optimal docking conformation and 3D structure of Lu complexed with the protein; and hydrogen bonds on the receptor surface of the optimal docking conformation between Lu and the protein. Reprinted from Ref. [133] with permission.

The second approach involves the use of modified exosomes as carriers. Common modification methods include extracting lipids as carriers for the reassembly or engineering of exosomes to endow them with special functions. Zhang et al. [132] designed alginate/polylysine-functionalized layer-by-layer ginger-derived lipid carriers (LbL-GDLVs) to target P-selectin and deliver the loaded drug to vascularized colon cancer. LbL-GDLVs loaded with doxorubicin (DOX) (LbL-GDLVs/DOX) significantly inhibited tumor growth and exhibited better therapeutic efficiency than free DOX. More importantly, LbL-GDLVs exhibited good biocompatibility, and the encapsulation of DOX in LbL-GDLVs significantly reduced the cardiac toxicity associated with free DOX and avoided colon cell resistance to free DOX. Additionally, Jiang et al. [133] developed plant exosome-like nanovesicles (Exos) derived from sesame leaves as delivery vehicles for lignans. The interaction with lignans was studied by molecular docking, and the stability and anti-inflammatory activity were evaluated *in vitro* (Fig. 8) [133].

**Fig. 9:**
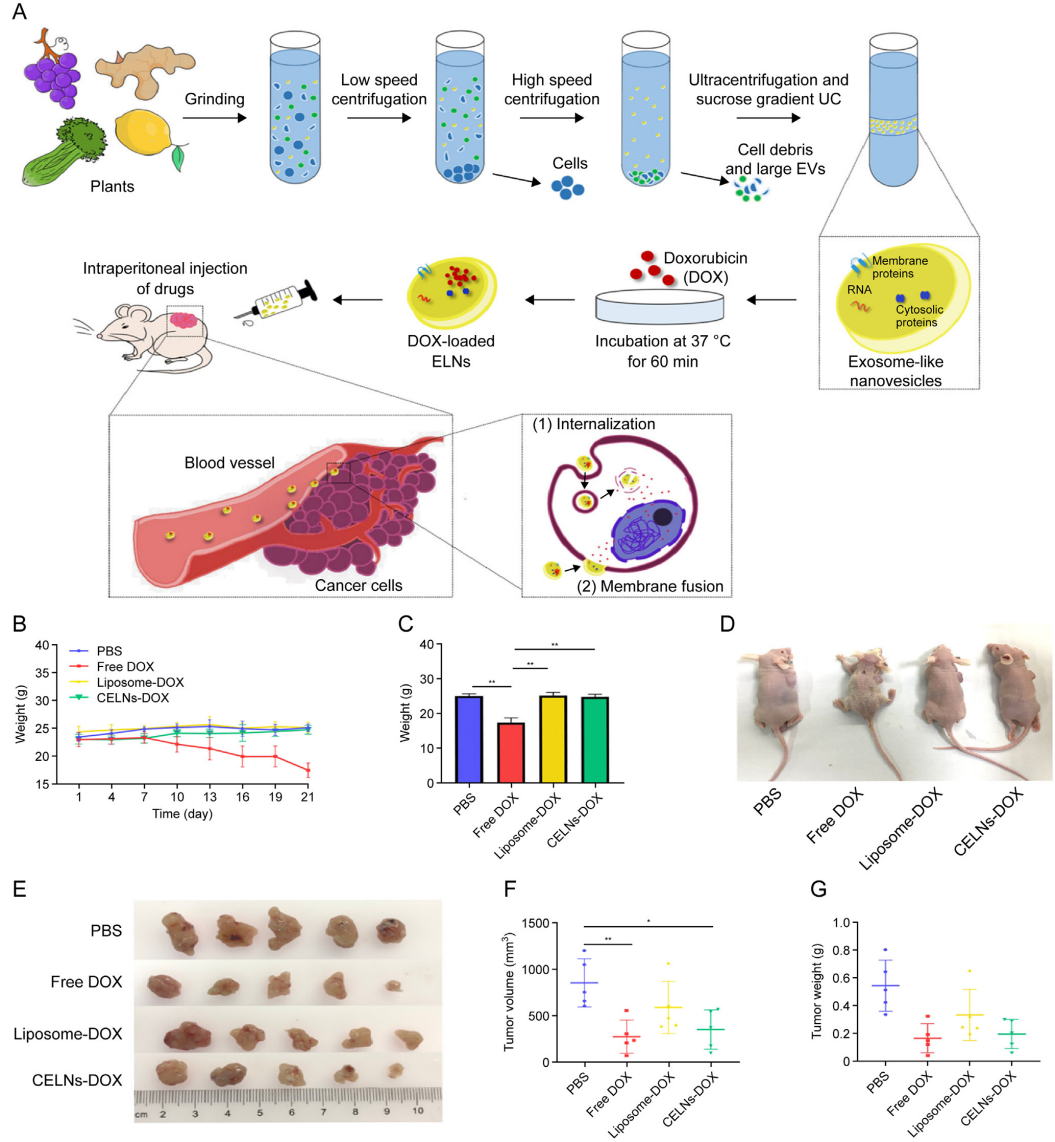
Celery-derived exosomes deliver doxorubicin (DOX) to tumor sites to exert therapeutic effects. (A) Schematic diagram of the isolation of exosome-like nanoparticles (ELNs) from lemon, ginger, grape, and celery. (B) Body weight changes in the mice from the four groups (phosphate-buffered saline (PBS), DOX, CELNs-DOX, and liposome-DOX). The term “1 day” indicates the first day after drug injection. (C) Comparison of the body weights of the four groups of mice at the end of the experiments. (D) Comparison of the body sizes of the four groups of mice at the end of the experiment. (E) Photographs of each group of tumors. (F, G) The size (F) and weight (G) of the tumors in each group were evaluated. Reproduced from Ref. [138] with permission.

Additionally, modifying PDEs to create optimal drug carriers is a commonly utilized strategy. Tang et al. [134] reported a novel HA1-functionalized aptamer-based DOX loading system using GNVs that specifically targets HER2^+^ breast cancer cells. The results indicated that this drug delivery system significantly increased drug delivery to the tumor tissue and increased its antitumor activity. Xiao et al. [135] described a biomimetic drug delivery system based on lemon-derived EV nanoparticles labeled with heparin-cRGD-EV-azithromycin (HRED). HRED was prepared by modifying EV surfaces with functional heparin-cRGD (HR) and then loading azithromycin (AZL). This method efficiently overcomes cancer multidrug resistance through energy dissipation and reduces ATP production induced by endocytosis. Zeng et al. [136,137] isolated aloe-derived nanocapsules (gADNVs) from aloe gel, which exhibited an appropriate size, good stability, and safety both *in vitro* and *in vivo* for indocyanine green (ICG) loading and melanoma treatment. This approach simplified the drug carrier preparation process, reduced production costs, extended drug storage time, and showed superiority over liposomes, providing a better option for developing economical and safe ICG delivery systems. Additionally, gADNVs were modified postinsertion with integrin-targeting peptides (Arg-Gly-Asp, RGD) to deliver ICG and DOX for the treatment of drug-resistant breast cancer. Furthermore, Lu et al. [138] encapsulated DOX into celery exosome-like nanovesicles (CELNs) to construct engineered CELNs (CELNs-DOX), which proved to be more efficient at treating tumors both in vitro and in vivo than traditional synthetic carriers such as liposomes. This method inhibited tumor proliferation while reducing the cardiotoxicity of DOX (Fig. 9) [138].

In nanodrug delivery systems, various carriers present distinct advantages and limitations. As emerging carriers for nanodrug delivery, PDEs have garnered significant attention because of their unique advantages, particularly in modern medical research, which emphasizes high efficiency, safety, and environmental sustainability [139,140]. Animal-derived exosomes are associated with immunogenic and pathogenic risks [141]. The use of exosomes from mouse or other mammalian cells may result an immune response triggered by the proteins or nucleic acids within these exosomes, leading to accelerated blood clearance and potentially reduced efficacy or adverse reactions [142,143]. In contrast, PDEs benefit from the safety of their natural origin. Exosomes isolated from tea leaves [144] have been shown to possess anti-inflammatory properties and reduce the risk of immunogenicity and pathogen transmission due to their plant-based origin. These properties make them safe and effective drug delivery vehicles for the treatment of inflammatory diseases.

Biocompatibility and safety concerns are often associated with synthetic nanoparticles, such as polylactic acid-hydroxyacetic acid copolymer (PLGA) nanoparticles, which are widely used for drug delivery but may cause local tissue damage or systemic toxicity with prolonged use [145]. In contrast, PDEs are highly biocompatible for various applications. For example, *Vitis vinifera* Kyoho-derived exosomes have been shown to effectively deliver multiple drugs, including metformin, DOX, and tamoxifen [146]. Compared with synthetic nanoparticles, PDEs not only enhance drug targeting but also exhibit superior biocompatibility and lower toxicity, making them promising candidates for cancer therapy.

Bionic vesicles (e.g., liposomes) often exhibit poor stability, with conventional liposomes being prone to fusion or leakage during storage, which compromises drug stability and the delivery efficiency [147]. For example, DOX-loaded liposomes have been found to accelerate drug release when stored at elevated temperatures or under refrigeration, thereby reducing therapeutic efficacy [148]. In contrast, PDEs exhibit relatively stable structures; for example, PDEs extracted from lemon can effectively encapsulate DOX and maintain its structural integrity at various temperatures and pH values. This stability prevents premature drug release before the drug reaches the target site, thereby enhancing treatment efficacy [135]. Although the specific mechanisms of selective packaging and release of drugs by PDEs and their interactions with target cells are not yet fully understood, the potential importance of PDEs in controlling physiological and pathological processes, as well as their role as drug carriers, suggests that a comprehensive and systematic analysis of the biomolecules in various PDEs will promote their application in drug delivery [149].

In current clinical trials, the use of animal-derived exosomes as diagnostic tools and therapeutic agents has increased in certain cases, but the clinical data concerning their use as drug delivery systems remain relatively limited. In contrast, examples of PDEs that have advanced to clinical trials are even rarer, with most studies focusing primarily on validating their physiological activities. For example, studies have documented the anti-inflammatory properties of grape-derived exosomes (NCT01668849), as well as the ability of ginger- or aloe-derived exosomes to ameliorate insulin resistance and chronic inflammatory conditions in patients with polycystic ovary syndrome (PCOS) (NCT03493984). Notably, in drug delivery applications, ginger-derived exosomes have achieved the effective targeted delivery of curcumin to the site of colon cancer via the oral route, revealing their therapeutic potential (NCT01294072). These preliminary clinical explorations not only highlight the potential efficacy of PDEs in treating specific diseases but also provide a theoretical foundation and experimental rationale for their future development as innovative drug delivery platforms. However, despite these advances, the clinical translation of PDEs in drug delivery continues to face several challenges, including but not limited to standardized production, large-scale manufacturing, and comprehensive safety and efficacy assessments. As research progresses and technology evolves, PDEs are expected to play an increasingly significant role in personalized medicine and precision therapies.

## 9 Summary

PDEs are emerging as novel types of biological particles with potential as targeted drug delivery carriers. These small vesicles, secreted by plant cells, contain a variety of bioactive molecules, including proteins, nucleic acids, lipids, and carbohydrates. Recent studies have highlighted several advantages of PDEs that make them ideal carriers for targeted drug delivery [150]. The benefits of using PDEs as drug delivery vehicles include low-cost sources from natural and abundant resources, the ability to obtain higher yields of exosomes through extraction methods, good stability and a longer circulation life, which contribute to the protection and prolonged action of drugs in the body; excellent biocompatibility and low toxicity, reducing immune reactions and toxicity issues associated with traditional carriers; and the ability to target specific organs or tissues by modifying their surface biomarkers, enhancing local therapeutic effects and reducing systemic side effects. Therefore, PDEs can serve as highly effective targeted delivery carriers for diverse drug delivery modalities, facilitating the precise administration of therapeutic agents to specific sites.

Research into PDEs as targeted drug delivery carriers faces challenges. The extraction and purification methods for PDEs require further optimization and standardization to facilitate large-scale production and application. Establishing robust quality control standards for PELNs is essential, ensuring consistency and reliability. For the same plant species, clear standards for the source of PDEs must be defined. Particularly in functional studies of herbal-derived PDEs, the selection of local herbs grown under specific environmental conditions and for consistent growth periods can increase the experimental reproducibility. Furthermore, a unified technical framework for the isolation and purification of PDEs should be developed promptly, alongside the identification of specific markers to characterize these exosomes. Increasing the stability of exosomes and improving their drug-loading efficiency remain significant challenges. Therefore, the components and mechanism of action of PELNs should be investigated in detail. Furthermore, when designing and developing PDEs as drug carriers, key considerations must include drug release kinetics, controlled release strategies, and interactions with target cells to ensure optimal therapeutic outcomes. As research on PDEs progresses, evaluating their safety and long-term effects becomes increasingly important. Following the verification of the safety and efficacy of PDEs, their clinical translational research should be actively promoted. In summary, PDEs hold broad application prospects as carriers for targeted drug delivery, yet further research and development are necessary to achieve their translation into clinical therapy.

## CRediT authorship contribution statement

**Haixia Shen:** Writing – original draft, Methodology, Conceptualization. **Shuaiguang Li:** Writing – original draft, Methodology, Conceptualization. **Liyuan Lin:** Software, Investigation. **Qian Wu:** Software, Investigation. **Zhonghua Dong:** Funding acquisition, Conceptualization. **Wei Xu:** Writing – review & editing, Funding acquisition, Conceptualization.

## Declaration of competing interest

The authors declare that there are no conflicts of interest.
